# Immune landscape and immunotherapy of hepatocellular carcinoma: focus on innate and adaptive immune cells

**DOI:** 10.1007/s10238-023-01015-2

**Published:** 2023-02-11

**Authors:** Xiaoqiang Gao, Shi Zuo

**Affiliations:** 1 Department of Hepatobiliary Surgery, Affiliated Hospital of Guizhou Medical University, No. 28, Guiyi Street, Guiyang, 550000 Guizhou China; 2 Guizhou Medical University, Guiyang, Guizhou China

**Keywords:** Hepatocellular carcinoma, Innate immune cell, Adaptive immune cell, Tumor immune microenvironment, Immunotherapy

## Abstract

Hepatocellular carcinoma (HCC) is responsible for roughly 90% of all cases of primary liver cancer, and the cases are on the rise. The treatment of advanced HCC is a serious challenge. Immune checkpoint inhibitor (ICI) therapy has marked a watershed moment in the history of HCC systemic treatment. Atezolizumab in combination with bevacizumab has been approved as a first-line treatment for advanced HCC since 2020; however, the combination therapy is only effective in a limited percentage of patients. Considering that the tumor immune microenvironment (TIME) has a great impact on immunotherapies for HCC, an in-depth understanding of the immune landscape in tumors and the current immunotherapeutic approaches is extremely necessary. We elaborate on the features, functions, and cross talk of the innate and adaptive immune cells in HCC and highlight the benefits and drawbacks of various immunotherapies for advanced HCC, as well as future projections. HCC consists of a heterogeneous group of cancers with distinct etiologies and immune microenvironments. Almost all the components of innate and adaptive immune cells in HCC have altered, showing a decreasing trend in the number of tumor suppressor cells and an increasing trend in the pro-cancer cells, and there is also cross talk between various cell types. Various immunotherapies for HCC have also shown promising efficacy and application prospect. There are multilayered interwoven webs among various immune cell types in HCC, and emerging evidence demonstrates the promising prospect of immunotherapeutic approaches for HCC.

## Introduction

The liver is a vital hub of macronutrient metabolism, lipid homeostasis, detoxification, and immune surveillance. In addition to its tolerance property toward antigens that are commonly encountered, the liver is an immune-active organ because of its responsibility to remove pathogens and gut-draining antigens from the systemic circulation. A healthy liver is primarily populated by leukocytes, including Kupffer cells (KCs), T cells, B cells, natural killer (NK) cells, and NKT cells [1, 2]. The tolerogenic and immune-rich environment of the liver maintains local and systemic homeostasis. Once the liver cancer has developed, the tolerogenic environment promotes tumor progression [3].

**Fig. 1:**
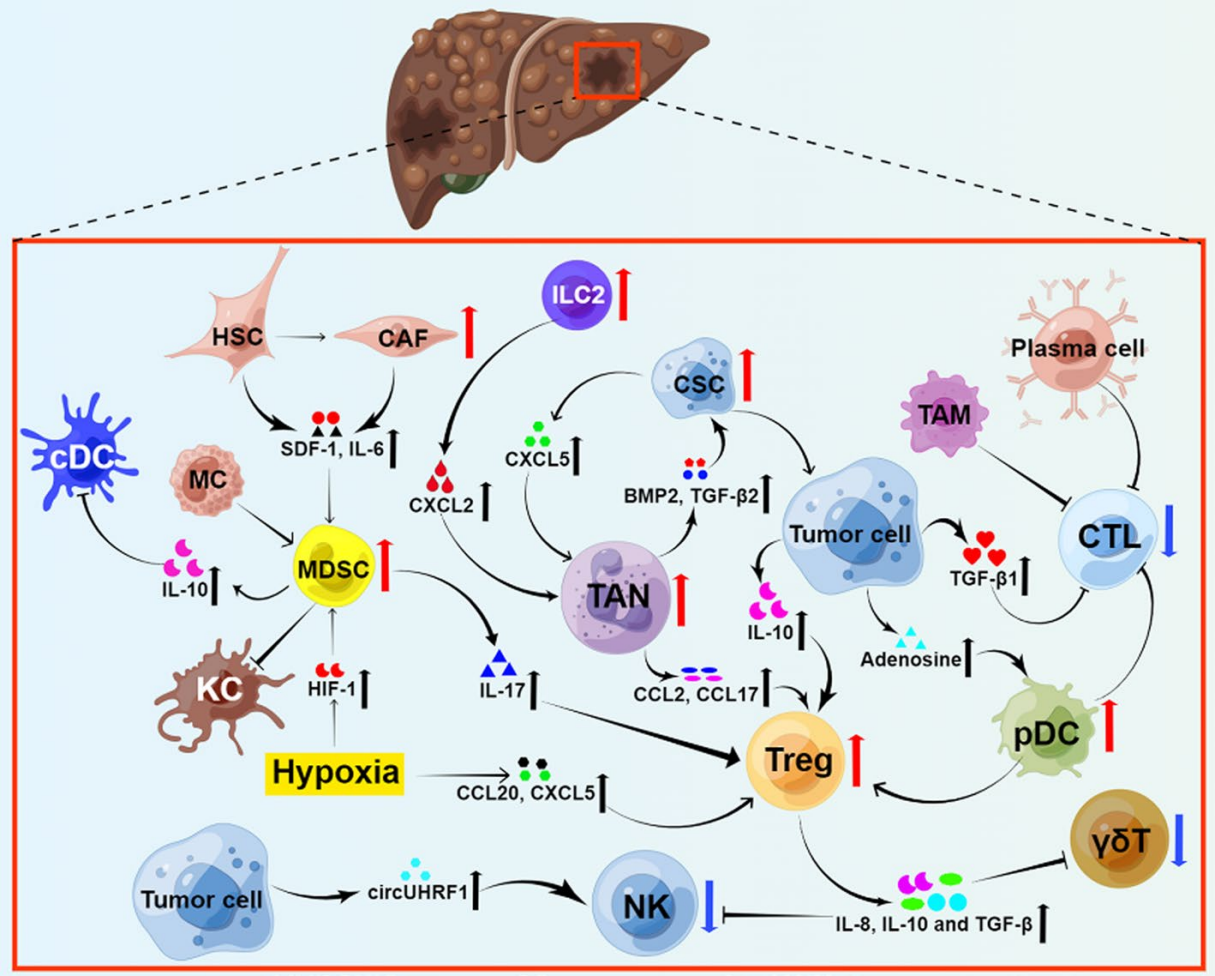
The tumor immune microenvironment of HCC. Tumor cells can evade host immune attack if HCC tumor antigens are not effectively recognized and presented by the immune system, or if the function of tumor-killing T lymphocytes is inhibited by suppressor cells or molecules in the tumor microenvironment. This figure illustrates the complex interactive network. In the figure, the vertically downward blue arrows and the vertically upward red arrows indicate the decrease and increase in the number of cells in HCC, respectively. The thick black arrows pointing upwards represent an increase in cell secretory products. Furthermore, arrows pointing to cells symbolize facilitative effects, while lines symbolize inhibitory action. TAN, tumor-associated neutrophil; MDSC, myeloid-derived suppressor cells; TAM, tumor-associated macrophages; Treg, regulatory T cell; pDC, plasmacytoid dendritic cell; NK, natural killer; KC, kupffer cell; MC, mast cell; CTL, cytotoxic T lymphocyte; CSC, cancer stem cell-like cell; cDC, conventional dendritic cell; HSC, hepatic stellate cell; CAF, cancer-associated fibroblast; ILC2, helper-like innate lymphoid cell 2

Hepatocellular carcinoma (HCC) is responsible for roughly 90% of all cases of primary liver cancer. Chronic hepatitis virus infections, alcohol misuse, metabolic syndrome, and several monogenic diseases are well-known risk factors for HCC. Most patients are diagnosed with HCC at an advanced stage since the disease is almost symptomless in the early stages. Advanced HCC used to be treated with transarterial chemoembolization (TACE) and tyrosine kinase inhibitors (TKIs), but these modalities do not significantly prolong the lifespan of patients [4]. However, immunotherapies are transforming cancer treatment [5]. Nivolumab and pembrolizumab, both anti-PD-1 drugs, have been used as second-line treatments for advanced HCC resistant to sorafenib [6, 7]. When compared to first-line sorafenib, the combination of atezolizumab (an anti-PD-L1) and bevacizumab (a VEGF blockade) provides superior outcomes and has become a new first-line treatment in advanced HCC [5]. Despite a significant paradigm shift in HCC treatment, immunotherapies still have limitations due to a lack of data on drug resistance and response prediction. Previous research has suggested that the tumor immune microenvironment (TIME) of HCC is significantly related to the prognosis and treatment response [8, 9]. This review summarizes the immunobiological features of HCC, with a focus on various innate and adaptive immune cells (shown in Fig. 1) in the tumor microenvironment (TME) and the cross talk among them. We also describe recent immunotherapeutic approaches and potential future directions.

## Innate immune cells in HCC

### Myeloid cells

#### Dendritic cells

Like dendritic cells (DCs) in other tissues, liver DCs mainly include conventional DCs (cDCs) and plasmacytoid DCs (pDCs) [10]. cDCs are the only cells in the body capable of activating naive T cells, whereas pDCs are inefficient antigen-presenting cells in the liver [11, 12].

In HCC, the proportion of LAMP3 + DCs that originated from cDC1 or cDC2 and are correlated with the malfunction of T lymphocytes is higher in tumor tissues than that in adjacent non-tumor tissues, and the fraction of pDCs in relapsed tumor samples is larger than that in primary tumor samples [13, 14]. Hypoxia is a common phenomenon in tumors. In severely hypoxic tumor regions of HCC, regulatory T cells (Tregs) and cDC2 can be attracted by CCL20 and CXCL5, and hepatoma cell-derived extracellular adenosine can recruit pDCs via the hypoxia-inducible factor (HIF)-1α/CD39/CD73 signaling pathway [15, 16]. HCC-produced α-fetoprotein (AFP) can impair biological metabolism of DCs by suppressing the expression of the metabolism regulators SREBP-1 and PGC1-α, mediate the dysfunction and apoptosis of DCs, and suppress the activation of NK cells by DCs [17–19]. In addition, in an in vitro study, tumor culture supernatants from HCC cell lines could impede the differentiation and maturation of monocyte-derived DCs, leading them to develop an immunosuppressive interleukin (IL)-10^high^IL-12^low^TNF-α^low^ cellular phenotype [20]. Tumor-associated DCs can induce tumor immune evasion via suppressing the functions of various T cell subsets and NK cells through multiple ligand-receptor pairs, including PD-1/PD-L1, and promoting the expansion of Tregs, which can mediate the loss of HLA-DR from DCs to maintain their tumor immunosuppressive activity, forming a positive feedback loop [13, 15, 16].

#### Neutrophils

Neutrophils are rich in the peripheral blood. The abundance of neutrophils depends on continuous supplementation through granulopoiesis in the bone marrow because of the limited lifespan of these cells. Neutrophils exhibit a strong effector response when they are recruited into inflammation sites [21]. In the past few decades, neutrophils have been regarded as a unique component that can promote the intercellular communication between tumor cells and TME [22]. Tumor-associated neutrophils (TANs) were confirmed to have different activation/differentiation states, including the N1 phenotype (anti-tumor) and the N2 phenotype (pro-tumor) [23]. N1 TANs have enhanced phagocytosis and migratory capacity, increased oxidative burst, and enhanced cytotoxicity for tumor cells. N2 TANs have inhibitory effects on T cells and are less cytotoxic to tumor cells [24].

In HCC, monocyte-derived CXCL2 and CXCL8 can recruit peripheral neutrophils to TME and sustain their survival [25], and tumor cells then educate the peripheral blood neutrophils to develop into CCL2 + or CCL17 + TANs via PI3K/Akt and p38/MAPK signaling pathways. As a result, TANs are distributed all over the tumor stroma. TANs can facilitate neovascularization and progression of HCC by the recruitment of macrophages and Tregs, which is related to the poor prognosis of HCC [26]. Activated neutrophils can form extracellular traps (NETs) under various inflammatory conditions [27]. NET formation is increased in HCC-derived neutrophils. The increased NETs can inhibit HCC cell death and enhance the invasiveness of HCC cells via activation of TLR4/9-COX2 signaling [28]. Furthermore, cortisol, which is mainly produced in males, can induce TGF-*β* expression in the liver, TGF-*β* is associated with TAN recruitment and the upregulation of some pro-tumor molecules, contributing to the gender disparity in HCC carcinogenesis [29]. Moreover, cancer stem cell-like cells (CSCs) have been confirmed to exist in various cancers, including HCC. CSCs are linked to the development and progression of HCC [30, 31]. TANs can increase the stem cell properties in tumor cells via the miR-301b-3p/LASMP/CYLD signaling pathway, and liver CSCs can recruit more TANs into the TME by secreting high levels of CXCL5 because of the hyperactive NF-*κ*B signaling. It is a positive feedback loop, ultimately fostering HCC progression [32]. A latest study found that cabozantinib (a TKI) combined with anti-PD-1 can promote the recruitment of N1 TANs in HCC and inhibit tumor progression [33]. Recently, some TAN-targeting drugs have undergone clinical testing and demonstrated some therapeutic benefits [34–36]. However, more investigation is required to ascertain the function of neutrophil phenotypes, particularly TAN phenotypes, in HCC.

#### Kupffer cells

KCs, which line the liver sinusoids, are the liver-resident macrophages. KCs are responsible for detoxifying blood that may contain harmful enteric pathogens or toxic digestive byproducts, as well as being involved in inflammatory processes, particularly in viral hepatitis and HCC [37]. There are two functionally opposite phenotypes of KCs in the healthy liver: classically (M1) and alternatively (M2) activated KCs, with a balance between them [38]. Besides KCs, there is another macrophage subtype in the liver, peripheral blood monocyte-derived macrophages (MoMFs) [39].

Even though KCs are the first line of defense against HCC cells, most studies have confirmed their pro-cancer roles in HCC, and TME-driven KC transition from M1 to M2 may be one important cause [38, 40, 41]. In mouse HCC tissues, myeloid-derived suppressor cells (MDSCs), an immunosuppressive cell, could inhibit the costimulatory molecule expression and the antigen-presenting function of KCs while increasing the expression of coinhibitory molecules in KCs, which could be another cause of the cancer-promoting KC formation [40]. In an HCC mouse model, M2-KCs were thought to be a key factor in tumor progression, and microRNA-206, which could promote M1-KC polarization, had been shown to increase the percentage of CD8 + T cells in HCC and suppress tumor growth [38]. Furthermore, based on a recent study, different types of TMEs were present in HCC, with distinct cell distribution patterns corresponding to different stages of hepatocyte dedifferentiation. The regional immunity of HCC was reversely regulated by KCs and infiltrating MoMFs with pro- and anti-tumor functions, respectively. In HCC mouse models, KC depletion could increase the intratumoral infiltration of MoMFs and improve the efficacy of anti-PD-1 antibodies, inhibiting tumor progression [41]. Mechanistically, KCs can suppress the tumor-killing toxicity of CD8 + T cells via B7-H1/PD-1 interactions or mucin domain-containing molecule-3 (Tim-3)/galectin-9 signaling pathways in HCC [42, 43]. In addition, KCs also play an important role in hepatocarcinogenesis [44–46]. Sympathetic nervous system-mediated activation of KCs, as well as autophagy-deficient KCs, can promote the tumorigenesis of HCC by increasing liver inflammation and fibrosis [44, 45]. TREM-1 (triggering receptor expressed on myeloid cells), the proinflammatory receptor on KCs, can also regulate the activation of KCs and the development of HCC [46].

#### Myeloid-derived suppressor cells

MDSCs exert immunosuppressive roles by releasing large quantities of active components [47]. Two major MDSC subpopulations, monocyte MDSCs (M-MDSCs) and polymorphonuclear MDSCs (PMN-MDSCs), were recently identified. Another subtype is early MDSCs (eMDSCs), which contain more progenitor cells that can differentiate into mature MDSCs [48]. In different conditions of TME, MDSCs may transform into DCs, neutrophils, or macrophages and conduct a variety of functions [49].

MDSCs are increased in the peripheral blood and tumor tissues of HCC patients, and the number of PMN-MDSCs in the peripheral blood exceeds that of M-MDSCs, which is associated with poor prognosis [50–56]. Hepatic stellate cells (HSCs), non-parenchymal hepatocytes, are activated when the liver is injured [57]. In HCC, activated HSCs can recruit MDSCs by producing the stromal cell-derived factor 1 and induce MDSC expansion via IL-6 signaling or the complement C3 pathway [58–60]. Cancer-associated fibroblasts (CAFs), which originate from HSCs, play pro-tumorigenic roles through their interaction with HCC cells, and CAF-derived cytokines can stimulate the generation of MDSCs [61, 62]. Furthermore, HIF-1 can promote MDSC accumulation via ectonucleoside triphosphate diphosphohydrolase-2, which can also inhibit MDSC maturation in HCC [63, 64]. In addition, chronic restraint stress-related β-adrenergic signaling in HCC can also recruit MDSCs through the CXCL5-CXCR2-Erk signaling pathway [65]. MDSCs can promote tumor progression via multiple pathways. MDSC-derived IL-10 has been shown to inhibit IL-12 production and DC activation, and MDSC-derived fibroblast growth factor 1 has been shown to activate CAFs, resulting in tumor growth [66, 67]. Moreover, M-MDSCs can suppress tumor immunity via the CXCL10/TLR4/MMP14 signaling, thereby increasing tumor recurrence after liver transplantation [68]. Endoplasmic reticulum (ER) stress, characterized by the accumulation of a large amount of structurally abnormal protein in the ER and the subsequent aberrant response, is confirmed to be associated with hepatocarcinogenesis [69, 70]. The increased PMN-MDSCs in HCC can impede T cell proliferation through the ROS/Arginase I pathway, which is mediated by ER stress [71].

#### Mast cells

Human mast cells (MCs) originate from hematopoietic stem cells. Mature MCs are observed in almost all tissues as hypergranular cells but are absent in blood. MCs can be classified into several subpopulations based on their properties. According to their tissue localization, MCs can be divided into two types: mucosal MCs and connective tissue MCs. Tryptase-expressing MCs (MCTs), tryptase and chymase-expressing MCs, and chymase-expressing MCs are classified based on their protease content. In terms of their roles in the disease, MCs are further categorized as inflammatory MCs, tumor-promoting MCs, and anti-tumor MCs [72, 73].

The number of MCs in tumor tissues is markedly lower than in peritumoral tissues in HCC [73–75]. The role of MCs in HCC is still debatable. On the one hand, MCs were demonstrated to promote the infiltration of MDSCs, which secreted IL-17, IL-17 then recruited Tregs, which produced IL-9; and IL-9, in turn, enhanced the immunosuppressive effect of MCs in HCC. There was a closed interaction loop among the three cell types that promoted tumor progression [76]. MCTs, the primary IL-17 producers, were reported to promote HCC angiogenesis [77, 78]. On the other hand, reduced intratumoral MCs in HCC were found to be related to a larger tumor size and a higher recurrence rate after liver transplantation, and by introducing microRNAs into tumor cells to inhibit the ERK1/2 signaling pathway, MCs could prevent HCC metastasis, showing the anti-tumor properties of MCs [75, 79]. MCs play different roles in HCC, possibly because different MC types have different functions, and researchers have not consistently classified or stratified MCs in depth. MCs and their products might also play different roles based on the different biological characteristics of various tumor cells. The granules and histamine produced by MCs could inhibit huh-6 cell growth and activate huh-6 cell apoptosis while enhancing HA22T/VGH cell proliferation [80]. Besides, higher peritumoral MC density was verified to be associated with a worse prognosis and an earlier recurrence of HCC [81].

### Innate lymphoid cells

#### NK cells

The liver is the organ with the largest number of NK cells in the human body [82]. Unlike other lymphocytes, NK cells promote cellular activation and target cells for clearance using the absence of self. NK cell function is under the dual control of activating and inhibitory receptors. The interaction of inhibitory receptors and the major histocompatibility complex (MHC)-I on normal hepatocytes inhibits the activation of NK cells. A common mechanism of NK cell activation is the MHC-I downregulation of malignant cells. Target killing of NK cells is executed by perforin, granzyme, and apoptosis-inducing ligands [83].

The number of NK cells in HCC tissues is decreased, and their functions are impaired, which is related to the unfavorable prognosis for HCC [84]. An elevated level of HCC cell-derived exosomal circular ubiquitin-like with PHD and ring finger domain 1 RNA (circUHRF1) in the peripheral blood of HCC patients correlates with the reduced tumor infiltration of NK cells. CircUHRF1 can also suppress NK cell cytotoxicity by increasing TIM-3 expression, which is mediated by the degradation of miR-449c-5p [85]. Similarly, high levels of plasma TGF-β in patients with HCC lead to metabolic and functional defects in circulating NK cells [86]. Furthermore, transmembrane 4 L six family member 5 (TM4SF5), which is abundant in HCC, inhibits the expression of NK cytotoxicity-stimulated membrane ligands SLAMF6, SLAMF7, and MICA/B on target HCC cells, resulting in the decreased number and functional impairment of NK cells [87]. Moreover, the epithelial cell adhesion molecule (EpCAM) is a CSC biomarker in HCC [88, 89]. CSCs with high EpCAM expression in HCC can resist NK cell killing by promoting the carcinoembryonic antigen-related cell adhesion molecule 1 expression [90]. Notably, there is also complex cross talk among various immune cells in the TIME. MDSCs and tumor-associated macrophages (TAMs) in HCC can suppress NK cell cytotoxicity through NKp30 on NK cells [91], and Tregs can impair the immune responses of NK cells by releasing immunosuppressive cytokines IL-8, IL-10, and TGF-*β* [92].

#### NKT cells

NKT cells, expressing both T cell receptor (TCR)-chains and NK cell markers (NKp46 and NK1.1), can recognize lipids and glycolipids with the presentation of CD1d [93, 94]. NKT cells are categorized into type I and type II cells based on their TCR rearrangement and glycolipid reactivity [95]. Activated type I NKT cells, which is also called invariant NKT (iNKT) cells, can affect downstream immune responses by secreting interferon (IFN)-γ and IL-4 [96, 97]. Type II NKT cells have more Vα rearrangement sequences, and they can promote tumor growth and metastasis with the activation of sulfatides [98, 99].

In HCC, the number of iNKT cells in the tumor tissues is obviously lower compared with the adjacent non-tumor tissues, and their low infiltration in tumors is associated with the advanced stages and vascular invasion [100]. HCC is closely linked to liver inflammation, and iNKT cells can mediate anti-tumor effects by suppressing the inflammatory response in the process of *β*-catenin-induced liver tumorigenesis [101]. A synthetic glycolipid called *α*-galactosylceramide (*α*-GalCer) can inhibit the growth of hepatoma cells in the murine liver by stimulating NKT cells, which in turn activate NK cells [102]. The adoptive transfer of a small number of NKT cells that have been ex vivo treated with HCC-derived antigens can suppress the tumor growth in HCC mice, and the effect is correlated with NKT cell number, STAT4 expression, and serum levels of IL-12, IFN-γ, and IL-4 [103]. Furthermore, DCs pulsed with tumor antigens have been shown to inhibit HCC progression by activating NKT and CD8 + lymphocytes and increasing IFN-γ production [104]. NKT cells can be recruited to the liver via CXCR6-CXCL16 in the murine HCC models. CXCR6-deficient mice have an apparent higher tumor burden and tumor progression after intraperitoneal injection of DEN due to a reduction of iNKT and CD4 + T cells in the liver [105, 106]. Bile acid metabolism usually has a certain impact on the immune system of the body. Primary bile acids can promote the recruitment of NKT cells to the liver by upregulating the expression of CXCL16, thereby inhibiting HCC progression, and secondary bile acids, which are converted from primary bile acids by relevant bacteria, play the opposite role [106, 107]. There are also subpopulations of NKT cells that are involved in HCC promotion. CD4 + iNKT cells, unlike their CD4- counterparts, can boost tumor growth by inhibiting the cytotoxicity of CD8 + T cells and promoting Th2 cytokine production in HCC [108, 109].

#### Gamma delta T cells

Gamma delta (*γδ*) T cells are the important fighters of the immune system, accounting for an average of 3.7% of CD3 + T cells in peripheral blood. Based on the TCR rearrangement, *γδ* T cells, the nonconventional T lymphocytes, can be divided into three groups: V*δ*1, V*δ*2, and V*δ*3 T cells. V*δ*2 T cells predominate in the blood, while the other two groups are abundant in tissues. They have a range of biological functions, including pathogen clearance, inflammation regulation, and tumor immunity [110, 111].

The infiltration of *γδ* T cells in HCC is significantly decreased compared with the peritumoral tissues, which may be due to their G2/M cell arrest and active apoptotic state in tumors, and the low infiltration was confirmed to be correlated with the poor prognosis of HCC [112, 113]. *γδ* T cells can secrete IFN-γ in the early stage, which is essential for anti-tumor immunity [114]. An in vitro study observed that *γδ* T cells could inhibit the viability of HCC cells, and histone deacetylase inhibitors and zoledronic acid could enhance the suppression [115]. In addition to the reduced number, the function of *γδ* T cells is also abnormal in HCC. The cytotoxicity of *γδ* T cells is suppressed by various factors. Tregs, as well as the imbalance between HSCs and *γδ* T cells, can impair the function of *γδ* T cells, [116, 117]. Furthermore, increased glutamine metabolism and decreased glucose and lipid metabolism in *γδ* T cells further exacerbate the cellular dysfunction [112]. Several studies have been conducted to improve the anti-tumor function of *γδ* T cells in HCC. Aminobisphosphonate has been shown to promote the expansion of peripheral Vδ2 T cells and inhibit tumor growth, providing a new approach for HCC therapy [118]. Vδ1 T cells engineered with soluble IL-15 and a glypican 3 (GPC-3)-receptor can efficiently destroy HCC cells [119]. Moreover, the cytotoxicity of *γδ* T cells in HCC can be restored after supplementation with normal Vδ2 T [112, 120].

### Other innate immune cells

Mucosal-associated invariant T (MAIT) cells, mostly located at mucosal sites and the liver, are innate-like cells. MAIT cells can recognize the antigens presented by MHC-I-related protein 1. When activated, they can perform anti-cancer activity by producing cytotoxic cytokines and substances [121]. In HCC, MAIT cells are greatly reduced in tumors compared to adjacent tissues, which may be attributed to the downregulation of CCR6, CXCR6, and CCR9 on tumor-educated MAIT cells [122–124]. In addition, MAIT cells exhibit pro-tumor properties, accompanied by immune checkpoint upregulation, decreased secretion of IFN-γ and IL-17, and increased IL-8 production [122, 123]. Co-administration of 5-OP-RU (5-(2-oxopropylideneamino)-6D-ribitylaminouracil, microbial riboflavin-derived antigen) and CpG (Toll-like receptor 9 agonist) can suppress the HCC progression by activating MAIT cells to release IFN-γ and cytotoxic substances, together with the accumulation of CD8 + T cells and NK cells in vivo [124].

Helper-like innate lymphoid cells (ILCs) consist of three subgroups: ILC1s, ILC2s, and ILC3s, which are functionally equivalent to Th1, Th2, and Th17 cells, respectively, and play a vital role in cancers [125, 126]. Hepatic ILC1s, including embryonic and postnatal subsets, most closely resemble NK cells in phenotype and function [127, 128], and they have been shown to play a suppressor role in the liver metastasis of tumors. ILC1s can suppress liver metastasis by limiting metastatic seeding and producing effector molecules, and their cytotoxicity can be calibrated by the activating receptor NKp46 on ILC1s [129–131]. ILC2s are the most clearly defined subset of helper-like ILCs, and Bcl11b and TGF-β are required for their development [132, 133]. In HCC, ILC2s are enriched in tumor tissues and are related to a poor prognosis. ILC2s can promote HCC progression through the CXCL2-neutrophil and IL-13-B cell signaling pathways [134, 135]. ILC3s express RORgt, which is essential for their function [136]. In an in vitro study, ILC3s exerted cytotoxicity against HCC cells mediated by TRAIL [137]. However, in a murine HCC model, ILC3s lacking the natural cytotoxicity-triggering receptor (NCR − ILC3s) promoted HCC progression by orchestrating the IL-23/IL-17 axis [138]. Similarly, a decreased serum short-chain fatty acid (SCFA) level due to the loss of the gut microbiota *Lactobacillus reuteri* in HCC mice promoted the release of IL-17A by ILC3s in tumors, which boosted tumor growth [139]. The contradictory conclusions of the in vivo and in vitro studies may be attributed to the remodeling of ILC3s by the TME, and further investigation is needed [140].

## Adaptive immune cells in HCC

T and B cells, the adaptive immune cells, are essential for HCC immunity. Their functions and immunotherapy applications in HCC have received a lot of interest [5]. Here, we provide a summary of recent developments.

Conventional T cells consist of CD8 + and CD4 + T cells, and the former outnumber the latter in the liver; CD8 + T cells are the main tumor-infiltrating lymphocytes that perform anti-tumor functions [141, 142]. CD4 + T cells, mainly including CD4 + T-helper (Th) cells and Tregs, are also crucial in tumor immunity [143]. In HCC, CD4 + effector memory T (Tem) cells and Tregs progressively grow in number from the adjacent non-tumor region to the leading-edge area (slightly) to the tumor core (significantly), whereas CD8 + Tem cells showed the opposite trend [144]. The inhibition of CD8 + T cell infiltration by TAMs and plasma cells may be one reason for the reduction of CD8 + T cells in HCC [145, 146]. CD8 + T cells also exhibit an exhausted state in HCC with a high expression of PD-1 and LAG-3, which is a gradual and ongoing process that peaks in TNM stage II tumors [147, 148]. Multiple factors contribute to the exhaustion of CD8 + T cells. Firstly, tumor endothelial cells, MDSC-like macrophages, M2 macrophages, and CAFs in HCC may facilitate the formation of CD8 + T cell exhaustion [149–152]. Secondly, TGF-β1 derived from HCC cells can upregulate PD-1 and CTLA-4 expression on T lymphocytes via the CaN/NFATc1 pathway and accelerate T cell apoptosis [153]. Thirdly, abnormal glycolytic flux and lactate synthesis, as well as alterations in S-adenosylmethionine metabolism, can promote the development of exhausted T cells [154, 155]. Tregs are immunosuppressive cells, and exosomal circRNAs and IL-10 produced by HCC cells can promote the stability and expansion of them via various signaling pathways [156, 157]. HBV infection is the leading cause of more than half of HCC cases worldwide, and the hepatitis B-induced IL-8 can drive preferential Treg polarization mediated by liver sinusoidal endothelial cell-derived TGF-*β* [158–160]. Furthermore, lactic acid can stimulate PD-1 expression on Tregs in HCC, leading to the enhanced immunosuppressive properties of Tregs [161]. It is noteworthy that Tregs recruited by circulating tumor cells (CTCs)-derived CCL5 in HCC can promote CTC metastatic seeding, which may contribute to the development of novel anti-metastasis treatment for HCC [162].

B cells are a type of antigen-presenting cell that can present antigens to T cells and produce specific antibodies. Plasma cells, which originate from B cells, are involved in antibody production. The function of B cells in HCC remains controversial [163]. Some studies showed that the proportion of CD19 + B cells was higher in HCC tissues than in paracancerous tissues, and an increased B cell number in tumors was not only associated with an advanced tumor stage but also promoted immune escape in HCC [146],[164]. Additionally, B cells in HCC tissues have somatic hypermutations and class-switched recombinations of the IgG phenotype that are not seen in normal liver tissues [165]. Since there is little direct communication between HCC cells and B cells, B cells may modulate tumor immunity through other mechanisms [145]. Regulating B cells (Bregs), originally defined as CD19 + CD24^hi^CD38^hi^ cells, play an immunosuppressive role in tumors, and the number of Bregs expressing IL-10 increases in HCC. Exosomal high-mobility group box 1 produced by HCC cells can promote Breg expansion via the TLR-MAPK signaling pathway, and TLR activation can also increase the expression of Bcl-6, which is required for HCC environmental factors to promote the formation of PD-1^high^ Bregs [146], [163, 166–168]. In addition, CXCR3 + B cells, which account for approximately 45% of infiltrating B cells in HCC, can induce M2b macrophage polarization via IgG pathways, and CCR6 + B cells can promote angiogenesis by interacting with CCL20 generated by HCC cells [169, 170]. However, Zhang and colleagues discovered that HCC exhibited a global alteration in the B cell compartments with a decrease in CD20 + B cells and all B cell subsets, and that high levels of CD20 + B cells, IgM + B cells, CD27 − B cells, naive B cells, and plasma cells in HCC were associated with improved clinical outcomes [171]. Different criteria for defining B cells and their subgroups may be the cause of the contradictory results, and more research is required in the future.

## HCC immunotherapies

**Fig. 2:**
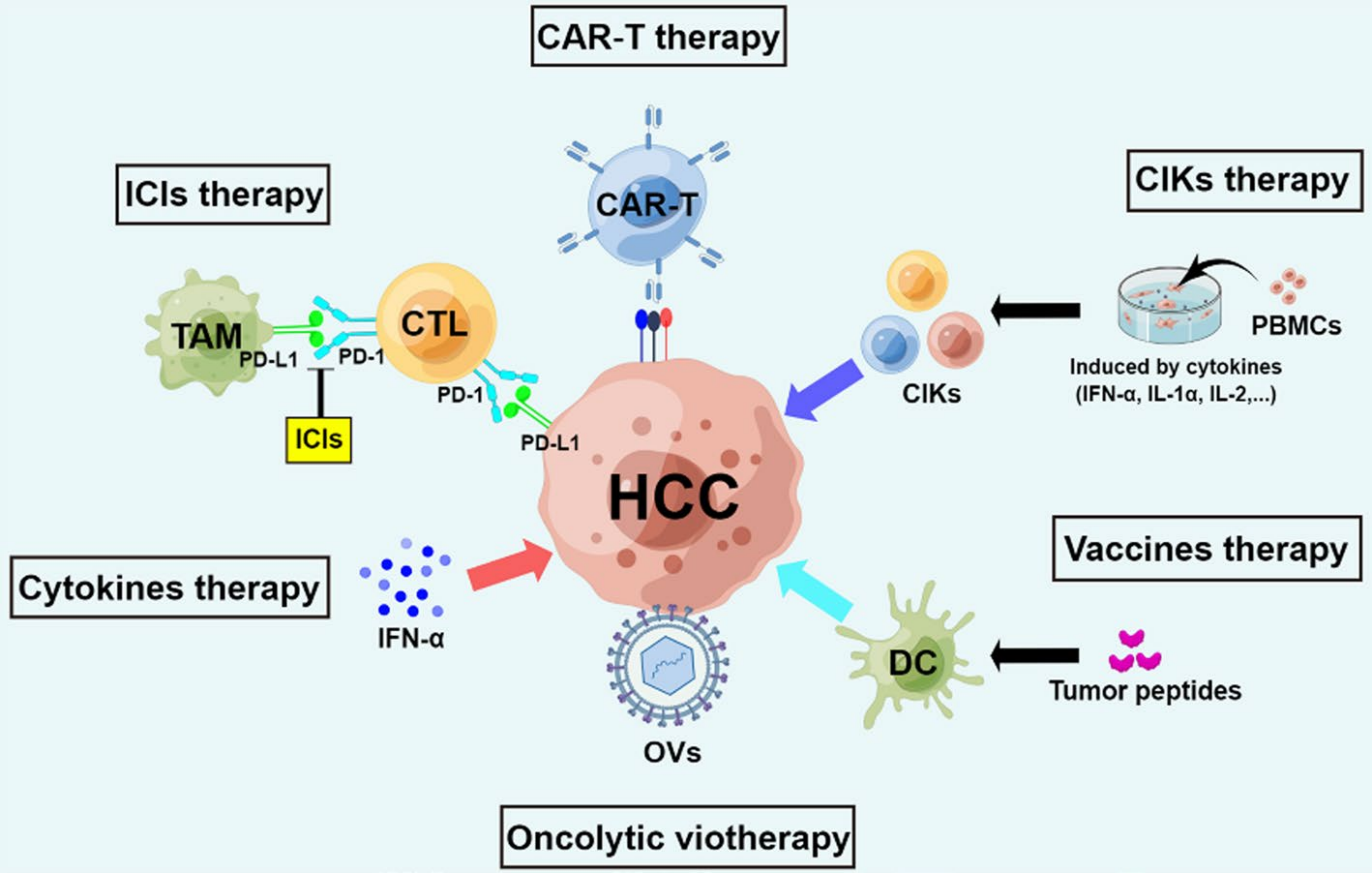
Recent immunotherapy concepts for HCC. CAR-T, chimeric antigen receptor-T cell; CIK, cytokine-induced killer; PBMC, peripheral blood mononuclear cell; IFN-α, interferon-α; IL-1α, interleukin-1α; DC, dendritic cell; OV, oncolytic virus; CTL, cytotoxic T lymphocyte; TAM, tumor-associated macrophage; ICI, immune checkpoint inhibitor; PD-1, programmed cell death-1; PD-L1, programmed cell death ligand 1

As HCC is an inflammation-related cancer, immunotherapy is a promising treatment option [172]. As a result of in-depth research into the TIME of HCC, new immunotherapy methods are constantly emerging. We summarize the most recent representative data from preclinical and clinical trials of immunotherapeutic strategies (shown in Fig. 2, Tables 1,2) for HCC and discuss their clinical application prospects.

**Table 1:** Summary of clinical trials of ICIs as monotherapy and in combination with other agents or treatments

Clinical trials	Phase	Disease stage	Patient numbers	Comparison arms	Results	Publication
*ICI monotherapy*						
CheckMate 040 (NCT01658878)	I/II	Advanced HCC	262	Nivolumab	Cohort 1 (dose escalation): ORR 15%, 9-month OS rate 66%, mOS 15 months Cohort 2 (dose expansion): ORR 20%, 9-month OS rate 74%	[6]
KEYNOTE-224 (NCT02702414)	II	Advanced HCC	104	Pembrolizumab	ORR 17%, mOS 12.9 months, 12-month OS rate 54%	[7]
NCT01008358	II	Advanced HCC	37	Tremelimumab	mOS 8.2 months, PR rate 17.6%, DCR 76.4%	[174]
KEYNOTE- 240 (NCT02702401)	III	Advanced HCC	413	Pembrolizumab vs. placebo	ORR 18.3%, mOS 13.9 months	[175]
NCT03163992	II	Advanced HCC	60	Pembrolizumab	Overall response rate 10%	[176]
CheckMate 459 (NCT02576509)	III	Advanced HCC	743	Nivolumab vs. Sorafenib	ORR 15%, mOS 16.4 months, 24-month OS rate 36.8%	[177]
*Combination ICI therapy*						
NCT02519348	I/II	Unresectable HCC	332	Tremelimumab + Durvalumab versus Tremelimumab versus Durvalumab	ORR 24%, mOS 18.7 months	[178]
CheckMate 040 (NCT01658878)	I/II	Advanced HCC	148	Nivolumab + Ipilimumab (3 dosing arms)	Arm 1: ORR 32%, Arm 2: ORR 31%, Arm 3: ORR 31%	[179]
IMbrave150 (NCT03434379)	III	Advanced/unresectable HCC	501	Atezolizumab + Bevacizumab versus Sorafenib	ORR 27.3%, mOS 19.2 months	[180]
NCT04297202	II	Resectable HCC	18	Camrelizumab + Apatinib	1-year RFS rate 53.85%, ORR 16.7% (based on RECIST)	[182]
NCT03006926	Ib	Unresectable HCC	104	Lenvatinib + Pembrolizumab	mOS 22 months, ORR 46% (based on RECIST)	[184]
NCT03299946	Ib	Locally advanced HCC	15	Cabozantinib + Nivolumab	80% successfully underwent margin negative resection	[185]
*ICIs in combination with other locoregional therapy*						
NCT01853618	II	Advanced HCC	32	Tremelimumab + ablation	mOS 12.3 months, 12-month PFS rate 33.1%	[186]

*HCC* hepatocellular carcinoma, *ORR* objective response rate, *OS* overall survival, *PR* partial response, *DCR* disease control rate, *RFS* recurrence-free survival, *RECIST* response evaluation criteria in solid tumors, *PFS* progression-free survival, *m* median

**Table 2:** Summary of clinical trials of other immunotherapies

Clinical trials	Phase	Disease stage	Patient numbers	Comparison arms	Results	Publication
*Adoptive cell therapy*						
NCT02541370	II	Advanced HCC	21	Anti-CD133 CAR-T cells	mOS 12 months, mPFS 6.8 months	[191]
NCT02395250 and NCT03146234	I	Advanced HCC	13	Anti-GPC3 CAR-T cells	3-year OS rate 10.5%, 1-year OS rate 42.0%, 6-month OS rate 50.3%	[192]
NCT01890291	II	Post-curative HCC	226	CIKs versus No treatment	5-year PFS rate 44.8%	[202]
*Cytokine therapy*						
NCT00149565	III	Post-curative HCC	268	IFNα-2b treatment versus No treatment	5-year OS rate 73.9%, 5-year RFS rate 44.2%	[208]
*Oncolytic virus therapy*						
TRAVERSE (NCT01387555)	IIb	Advanced HCC	129	Pexa-Vec plus BSC versus BSC	mOS 4.2 months	[236]

CAR-T cells, chimeric antigen receptor-T cells; GPC3, Glypican-3; CIK, cytokine-induced killer; IFN*α*-2b, interferonα-2b; Pexa-Vec, Pexastigmogene devacirepvec; BSC, best supportive care

### Immune checkpoint inhibitor therapy

Immune checkpoints are specific membrane molecules that are related to immune escape in cancers. There are many studies on the major immune checkpoints, such as CTLA-4, PD-1, and PD-L1, for immunotherapy [173]. Tremelimumab is a CTLA-4 blockade. A preliminary clinical study found that HCC patients treated with tremelimumab had a median overall survival (OS) of 8.2 months and a disease control rate of 76.4% [174]. The results of the KEYNOTE-240 trial showed that the median OS and progression-free survival (PFS) with pembrolizumab were 13.9 months and 3.0 months, respectively, when it was studied as a second-line therapy [175]. Additionally, pembrolizumab had a 10% overall response rate in HCC patients who failed sorafenib treatment in a recent study [176]. What is more, the median OS with single-agent nivolumab as first-line therapy for HCC was 16.4 months in the CheckMate-459 trial [177]. Although these single ICI therapies have certain anti-tumor effects in HCC patients, the efficacy is still unsatisfactory, which may be associated with the complex TIME of HCC.

As multiple mechanisms are involved in the tumorigenesis and progression of HCC, combining ICIs with other drugs or treatments may be a promising approach for HCC treatment. First, the combination of two ICIs targeting different immune checkpoints is effective for HCC therapy [178, 179]. The objective response rate (ORR) of tremelimumab combined with durvalumab (an anti-PD-L1 monoclonal antibody) in HCC patients was 24.0% in a clinical trial [178]. Likewise, another study found that nivolumab plus ipilimumab (an anti-CTLA-4 agent) showed a long-lasting response and a promising response rate in HCC patients who had received sorafenib treatment before [179]. Second, ICIs combined with anti-angiogenic drugs are effective against HCC [180–182]. VEGF overexpression in HCC is linked to a high density of tumor blood vessels [183]. In the IMbrave-150 trial, patients with atezolizumab (a PD-L1 inhibitor) plus bevacizumab (a VEGF blockade) had an ORR of 30% and a median OS of 19.2 months, and the combination therapy also exhibited good efficacy for HCC patients in South Korea in the real world [180, 181]. Furthermore, camrelizumab (an anti-PD-1 drug) in combination with apatinib (a VEGFR2 monoclonal antibody) demonstrated favorable perioperative outcomes in patients with surgically resectable HCC [182]. Third, ICIs combined with TKIs such as lenvatinib and cabozantinib are an effective treatment option [184, 185]. In a phase Ib study, the combination treatment of pembrolizumab and lenvatinib had an ORR of 46.0% and a median OS of 22 months in HCC patients [184]. Moreover, neoadjuvant therapy of nivolumab combined with cabozantinib could convert locally advanced HCC to resectable disease with the promotion of anti-tumor immunity [185]. At last, ICIs in combination with locoregional treatments, such as ablation, TACE, and stereotactic body radiotherapy, are available for HCC treatment [186–188].

### Adoptive cell therapy

There are two cell types of adoptive cell therapy (ACT) that are applied in the preclinical and clinical studies of HCC: genetically modified lymphocytes and cytokine-induced killer cells (CIKs). The objective of genetic modification of lymphocytes is to equip them with chimeric antigen receptor (CAR) to better target tumor-specific antigens [189]. GPC-3, a carcinoembryonic proteoglycan on the tumor cell membrane, and CD133, an endothelial progenitor cell marker, are two popular immunotherapy targets in recent HCC research [190, 191]. GPC-3-targeted CAR-T cells have been proven to have good safety and efficacy in the therapy of HCC, and GPC-3-CAR-T cells with the co-expression of IL-15 and IL-21 demonstrated superior cell proliferation and anti-tumor ability [192–194]. 8F8 is a low-affinity, GPC-3 specific antibody. 8F8-targeted CAR-T cells can withstand exhaustion and maintain anti-tumor effects in tumor lesions for a long time [195]. Similarly, CD133-targeted CAR-T cells show significant cytotoxicity against HCC cells [191]. What is more, GPC-3-targeted CAR-NK cells/Vδ1 T cells also exhibit robust anti-tumor activity in HCC [119, 196].

CIKs, exhibiting the phenotype and cytotoxicity of T cells and NK cells, contain multiple cell subsets [197]. CIKs can identify and destroy HCC CSCs through NKG2d-ligand recognition, thereby inhibiting HCC progression [198]. There is a cross talk between CIKs and MDSCs in HCC, and suppressing MDSCs can enhance the cytotoxicity of CIKs [199]. Adjuvant immunotherapy with CIKs in HCC patients could improve prognosis and quality of life when combined with radical therapy, TACE, or radiofrequency ablation [200–202], and receiving CIK therapy after curative treatment was proved to be cost-effective due to the reduced recurrence and prolonged survival of HCC [203]. In addition, PD-L1 and PD-1 might be used as two biomarkers to guide CIK treatment in HCC because high PD-L1 expression and a high infiltration of PD-1 + lymphocytes in HCC were all found to be correlated with good efficacy for CIK therapy [204, 205].

### Cytokine therapy

In terms of cytokine therapy, IFN-*α* has received more attention and is still being studied. IFN-α is a type I IFN with immunostimulatory and anti-vascular properties. Recombinant IFN-*α* is the first immunotherapy for human cancers [206]. According to some studies, IFN-α administration did not significantly improve the survival of HCC patients, and adjuvant IFN-α therapy after resection had no effect on RFS in HCC patients [207, 208]. Some studies, however, found that IFN-*α* could provide a significant benefit in both OS and RFS for HCC patients undergoing curative surgery, and that IFN-*α* therapeutic response could be predicted by hepatic retinoic acid-inducible gene-I and tetratricopeptide repeats 3 [209–211]. As a result of the heterogeneity of treatment responses, INF-α is not widely used in the clinical practice of HCC therapy.

Recent studies have confirmed that IFN-α treatment can recruit the cytotoxic T cells in murine HCC models by remodeling glucose metabolism, as well as promote the infiltration of cytotoxic CD169 + macrophages and M1-like macrophages, and that combined treatment with ICIs or sorafenib has synergistic anti-tumor efficacy [212–214]. These findings open a new avenue for the future use of IFN-α in the HCC therapy.

### Therapeutic vaccines

The main purpose of using cancer vaccines is to generate specific anti-tumor responses with strong potency. Classic cancer vaccines include peptide vaccines and antigen-pulsed DC vaccines. Tumor-associated antigens (TAAs), such as AFP, GPC-3, and telomerase, are common targets for HCC-specific peptide vaccines [215]. Recent research has primarily concentrated on GPC-3-related HCC vaccines. Because of the elevated density of peptide-specific cytotoxic T lymphocytes (CTLs) in the TME, HCC patients with high GPC-3 expression in tumor tissues and/or high content of GPC-3 in plasma have a high response rate and a good prognosis for the GPC-3 vaccine [216]. H8B-BsAb, a novel tetravalent bispecific anti-GPC-3 antibody, showed significant anti-tumor effect in a xenograft mouse model of HCC, and may be a potential candidate for HCC therapy [217]. Aside from antibodies, the GPC-3-modified molecules perform well against HCC [218–220]. Besides, other peptide vaccines, such as the aspartate-hydroxylase vaccine, the HA (the fusion of high-mobility group nucleosome binding protein 1 and AFP) vaccine, and the VEGF vaccine, also have promising applications in the treatment of HCC [221–223].

DC vaccines have been widely used in the treatment of various cancers, including HCC [224, 225]. A meta-analysis found that DC vaccines had a higher ORR and longer median OS and PFS compared to peptide vaccines in HCC treatment [226]. Many studies on novel DC vaccines have recently emerged, laying a solid theoretical foundation for the development of effective DC vaccines in the treatment of HCC. CD40L on activated CD4 + Th cells can communicate with CD40 on DCs to promote the secretion of Th1 cytokines by DCs, and the CD40L-DCs were confirmed to improve the anti-tumor activity of the AFP-DC vaccine in an orthotopic HCC mouse model, and the combination of them could significantly suppress tumor progression, accompanied by a robust Th1-shift in the TME and increased tumor cell apoptosis [227]. Furthermore, CSC/DC fusion cells could promote CTL accumulation in HCC and enhance anti-tumor immunity in animal experiments [228]. Recently, a DC-based nano-vaccine that consisted of silicon phthalocyanine dichloride, Fe(III)-captopril, and the exfoliated membrane of mature DCs stimulated by specific H22 cell neoantigens appeared, and it was proved to induce the activation and proliferation of neoantigen-specific T cells, as well as convert N2-type neutrophils to N1-type neutrophils in H22 tumors [229]. In addition, the combination of DC vaccines with other treatments such as ICIs, CIKs, and ACT demonstrates robust anti-tumor efficacy and may be a promising treatment strategy for HCC [230–233].

### Oncolytic virus therapy

Oncolytic viruses (OVs) can spread through tumor tissues, replicate selectively in cancer cells, and annihilate them without impairing normal cells [234]. The most common OVs for HCC therapy in preclinical and clinical studies are vesicular stomatitis virus and adenovirus. Pexastigmogene devacirepvec (Pexa-Vec) is a main OV that is currently being investigated in HCC; however, the efficacy of Pexa-Vec in clinical trials is disappointing [235, 236]. Nonetheless, studies involving other OVs are ongoing and have yielded promising results in animal models of HCC.

The influenza virus (IV), an RNA virus, has been identified as a potentially effective oncolytic agent. In HCC mouse models, recombinant IV with PD-1 antibody or CTLA4-specific scFv could activate anti-tumor immunity [237, 238]. Vaccinia virus, which is a double-stranded DNA virus, could enhance anti-HCC effects when carried with IL-24 or *Aphrocallistes vastus* lectin [239, 240]. T cell immunoglobulin and ITIM domain (TIGIT) expressed on activated NK and T cells is a key checkpoint molecule. The poliovirus receptor (PVR) is the cognate ligand of TIGIT [241]. In a recent study, an adenovirus expressing a PD1-PVR fusion protein could inhibit tumor growth mediated with CD8 + T cells in a mouse model of H22 ascites HCC, and it showed a better effect when combined with fludarabine [242]. However, OVs also have some drawbacks for application, mainly including non-specific distribution to organs and the generation of neutralizing antibodies [243, 244]. To overcome these shortcomings and improve the therapeutic efficacy of OVs, a research team exploited a polygalactosyl-b-agmatyl copolymer-coated oncolytic adenovirus, which displayed enhanced infectivity and tumor cell killing activity in vitro and provided a theoretical basis for the effective treatment of HCC with OVs in the future [245].

## Conclusions

As indicated by immunological classification, HCC consists of a heterogeneous group of cancers with distinct etiologies and immune microenvironments. There are multilayered interwoven webs among various immune cell types in HCC, and different stages of HCC are often accompanied by phenotypic changes of different immune cells in the TME, requiring thoughtful treatment design to ensure the success of immunotherapy. Emerging preclinical and clinical evidence demonstrates the promising prospect of immunotherapeutic approaches for HCC. With the advent of cutting-edge technologies such as single-cell approaches and multiplex histological analysis that preserve spatial information, it is possible to gain a better understanding of HCC immune status and discover new immunotherapy targets and patient-tailored approaches.

## Data Availability

The corresponding author will respond to reasonable requests for the datasets used in the current work.
